# A rare case of posterior reversible encephalopathy syndrome following posterior fossa ependymoma resection a surgical case report

**DOI:** 10.1016/j.ijscr.2024.110514

**Published:** 2024-10-24

**Authors:** Chrystal Calderon, Devindra Ramnarine, Patrick Knight, Robert Ramcharan

**Affiliations:** a Eric Williams Medical Sciences Complex, Mount Hope, Trinidad and Tobago; b Port of Spain General Hospital, Port of Spain, Trinidad and Tobago

**Keywords:** Posterior reversible encephalopathy syndrome (PRES), Neurosurgery, Posterior fossa tumor, Ependymoma, Blood pressure, Case report

## Abstract

**Introduction:**

Posterior reversible encephalopathy syndrome (PRES) is a rare complication following surgical intervention, with varied neurological manifestations. The inherent pathophysiology is diverse and risk factors include certain medical co-morbidities.

**Presentation of case:**

A previously well 24-year-old female, presented with signs of elevated intracranial pressure, with further investigations highlighting a posterior fossa tumor. She was scheduled for resection of this intracranial lesion and the surgical procedure was uneventful. However, moderate but significant labile increases in blood pressures were noted intra- and post- operatively. Following surgery, a clinical presentation of limb weakness and gaze deviation was observed, leading to investigative imaging demonstrating PRES. She was treated expeditiously by a multi-disciplinary team. There was complete resolution of her symptomology once the underlying cause was identified.

**Discussion:**

PRES is not a typical complication of a neurosurgical patient. Moreso, in a young patient without any medical comorbidities. Deviation of her blood pressures from the normal lead to the formation of vasogenic edema along the cerebral hemispheres. The manifestation of this clinically made it arduous to pinpoint a definitive diagnosis. With the aid of different specialists, a diagnosis was clenched, and treatment was successfully implemented.

**Conclusion:**

The major learning point of this case history is the recognition of alterations from a patient's baseline vital signs (blood pressure) during and following surgical procedures. Additionally, the resultant consequences of these deviations, which may manifest as rare neurological conditions, such as PRES. The importance of a multi-disciplinary team in the management of this case was paramount.

## Highlights

• PRES is rare complication following neurosurgical intervention.
• A high level of clinical suspicion is warranted in diagnosing these atypical cases.
• Avoidance of PRES relies on early detection of labile blood pressure during surgery.
• A multi-disciplinary team approach is essential to successful management.

## 1 Introduction

Posterior reversible encephalopathy syndrome (PRES) was first defined in 1996 by Hinchey et al. An at risk subset of patients who may be hypertensive, immunosuppressed or renal impaired was described [1]. Since then, efforts have been made to comprehend the underlying pathophysiology of this clinical entity. Two proposed theories are the cytotoxic and vasogenic theories [2,3].

All age groups are affected but PRES seems to be more common in the adult population, with a female predilection [4]. The spectrum of clinico-radiological features in this pathological entity makes it difficult to distinguish from more common diagnoses. Post-operative PRES outlines a different, infrequent group of patients, especially in the neurosurgical field. This is a unique case of post-operative PRES following a posterior fossa ependymoma resection, in a young female patient with no underlying comorbidities.

This work has been reported in line with the SCARE criteria [5].

## 2 Presentation of case

A 24-year-old female presented with a 1-year history of initial headaches, with associated nausea and vomiting. There was accompanying gait imbalance, dysphagia and weight loss. Of note, there was no significant past medical or drug history. On examination, her Glasgow coma scale (GCS) was 15, and an ataxic gait was observed with MRC grade 5 power throughout, hyperreflexia and a positive Hoffman's sign. A mild cranial nerve (CN) IX and X palsy was demonstrable.

**Fig. 1:**
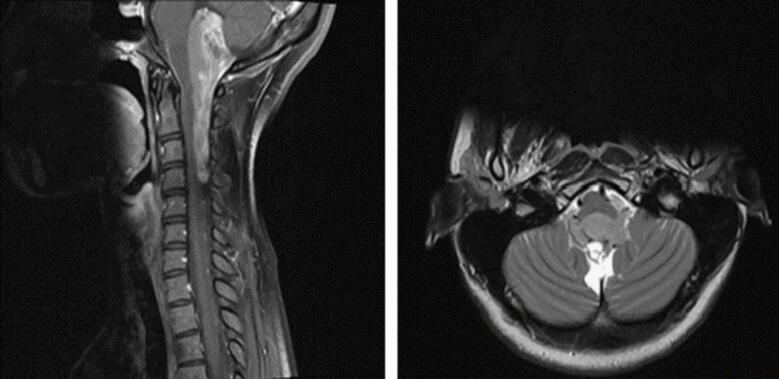
MRI brain and cervical spine demonstrating a posterior fossa tumor extending downward past the foramen magnum to the C4 vertebral body, on T1 weighted post gadolinium sagittal view and T2 weighted axial view, respectively.

Computed tomography (CT) brain showed the presence of obstructive hydrocephalus and an undefined mass within the fourth ventricle. Ventriculoperitoneal shunt placement was performed urgently. Magnetic resonance imaging (MRI) brain demonstrated a posterior fossa lesion extending past the foramen magnum down to the cervical level —C4 (Fig. 1). The patient was counselled and scheduled for a suboccipital craniotomy, C1 laminectomy and C2-C4 laminoplasty by the consultant neurosurgeon. Her pre-operative systolic blood pressure (SBP) ranged between 102 mmHg–110 mmHg. Approximately 2 h intra-operatively, a surge in SBP was noted by anesthetic team of 140 mmHg. This was not noted to be significant and was not managed intra-operatively. The surgical procedure was otherwise uneventful, with a gross total resection achieved.

**Fig. 2:**
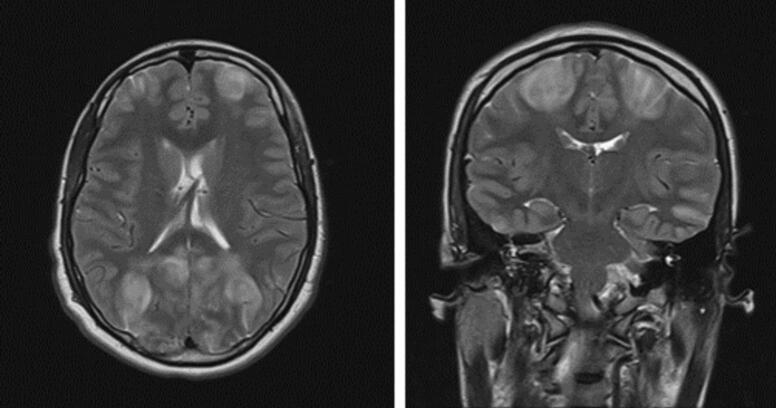
MRI Brain T2 weighted imaging (axial and sagittal views) demonstrating hyperintensities, in keeping with cerebral edema, involving the frontal and occipital lobes bilaterally.

The patient was kept intubated and sedated post- operation and admitted to intensive care unit (ICU). The following day, sedation was weaned, and SBP remained at approximately140 mmHg. GCS was 10 T, however power was MRC grade 3 in bilateral upper and lower limbs with a horizontal conjugate gaze palsy to the right. There was a subsequent failed trial of extubation. Post-operative CT brain depicted no significant intracranial findings. Day 2 of ICU admission, examination showed attenuation of power in upper and lower limbs, MRC grade 2. MRI brain demonstrated the presence of edema, more predominantly along the frontal and occipital lobes of the brain, greater along right hemisphere, in keeping with PRES (Fig. 2).

**Fig. 3:**
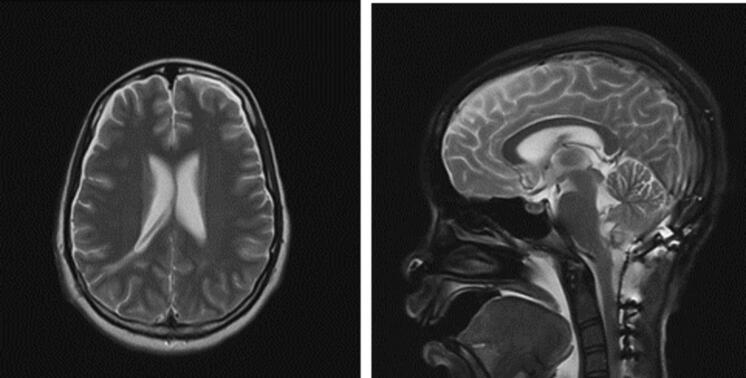
MRI brain T2 weighted (axial and sagittal views) demonstrating resolution of cerebral edema along bilateral cerebral hemispheres.

A multidisciplinary team was consulted, and aggressive medical management of the patient's SBP commenced, with a goal SBP of 100 mmHg. On day 7, improvement was noted clinically with an increase power MRC grade 4 throughout, gaze palsy resolution, but persistent diminished gag reflex. A tracheostomy and gastrostomy tube were inserted to aid in management. MRI brain done around this time showed resolution of edematous changes, with ongoing anti-hypertensive medications use (Fig. 3).

Histopathology results demonstrated grade II ependymoma. At approximately one year post operation, the patient is free of tracheostomy and gastrostomy tubes, ambulating without assistance, and with resolution of all neurological deficits.

## 3 Discussion

PRES presents as a plethora of neurological and radiological features [3,6]. These stem from a myriad of pathologies including labile blood pressures, autoimmune conditions, cytotoxic agents, and renal disease [6,7]. Labile elevations in blood pressures in this context refers to rapidly altering or inconsistent blood pressures but may still be within a normal range. The underlying pathophysiology of this condition is intricate in nature. It arises from injury to the vascular endothelium leading to a responsive release of cytokines. A sudden and significant elevation of blood pressure in the presence of an inappropriate cerebral autoregulatory response result in a state of hyper-perfusion. There is breakdown of the blood-brain barrier and subsequent vasogenic edema. The posterior circulation of the brain is usually prone secondary to a relatively deficient sympathetic innervation in this region. This hypertension hyper-perfusion theory is one of the explanations for this pathological event [3]. One of the major risks for this event is an abrupt increase in the blood pressure, even in a previously normotensive patient, which was seen in this case study.

The clinical presentation of PRES can be very non-specific with a varied timeline of events, including its resolution. Symptoms include features of encephalopathy such as cognitive decline and stupor, seizures, headaches, visual obscurations, and other neurological deficits [3,6]. PRES can be extremely difficult to diagnose in the post-operative period based solely on clinical findings, especially in an intubated patient following a major neurosurgical intervention, where other factors may be causative [8]. The possible differential diagnoses in this situation are extensive.

The diagnosis is based on radiological imaging, more specifically magnetic resonance imaging (MRI). The modalities that are most helpful in delineating the presence of vasogenic edema are T2- weighted imaging and fluid attenuated inversion recovery (FLAIR) sequences. Bilateral, symmetric, vasogenic edema is typically depicted involving the parieto-occipital regions in 98 % of cases, although variations may exist and are reliant on the severity of the pathological process [3,9]. In some cases, PRES may also be associated with underlying hemorrhage [3,4]. While in others, a holo-hemispheric watershed pattern may be distinguishable [9,10]. Initial post-operative radiological findings in the case patient demonstrated only mild edematous changes, that became more discernable, on repeat imaging, over the following days.

This indexed case depicted a complex posterior fossa tumor resection, with cranial nerve deficits prior to surgery. The possible peri-operative complications that may occur in this delicate region of the brain are numerous. However, the patient's presentation of PRES was not one of the primary considerations. We believe that this was secondary to an abrupt spike in the blood pressure intra- operatively, which was not seen to be clinically significant at the time. The exact cause of this blood pressure surge was not clear. Unfortunately, its impact was quite significant.

Less than fifty (50) cases of post-operative PRES have been reported in the literature, with even fewer following a posterior fossa ependymoma resection [11]. Cranial neurosurgery and maxillofacial surgery did represent the greater portion of cases identified in a literature review. Additionally, 36 % of patients in this analysis had no underlying risk factors for developing PRES [11]. It has been postulated that the postoperative PRES patient may be more susceptible to this condition at a lower blood pressure than the general population [12]. This case required a multi-disciplinary team to aid in the diagnosis and management of this young patient with no comorbidities. The clinical presentation here was not in keeping with the expected post-operative findings for the surgery performed. The appropriate post-operative supportive care was implemented which included avoidance of any alterations to the patient's normal blood pressure, with a focus on pain control, anti-hypertensive medications, and identifying any causative factors that may lead to labile blood pressures.

Although PRES is a relatively self-limiting condition once managed aptly, it has been associated with mortalities. Fortunately, the overall prognosis is satisfactory, with reversal of both clinical and radiological findings [4,8,11]. This was achieved in this case with complete resolution of her clinical findings over the course of the weeks following surgery. We recommend close monitoring of blood pressures in neurosurgical patients, even the unexpected, healthy cases.

## 4 Conclusion

Post-operative PRES following a complex resection of a posterior fossa ependymoma is a rare case. Hence, identifying the diagnosis in this patient required a multidisciplinary team approach. An element of clinical suspicion was pivotal in this case, as her post-operative findings did not match the expected outcomes for her surgical case. There is no specific treatment for PRES, and supportive measures are usually sufficient for a complete recovery in these cases.

## Author contribution

Chrystal Calderon- Conception and design, drafting the article, project administration, supervision, validation, revising it critically for important intellectual content, final approval of the version to be submitted.

Patrick Knight- Analysis and Interpretation of Data, revising it critically for important intellectual content, final approval of the version to be submitted.

Robert Ramcharan- Analysis and Interpretation of Data, revising it critically for important intellectual content, final approval of the version to be submitted.

Devindra Ramnarine- Analysis and Interpretation of Data, revising it critically for important intellectual content, final approval of the version to be submitted.

## Consent

Written informed consent was obtained from the patient for publication of this case report and any accompanying images. A copy of the written consent is available for review by the Editor-in-Chief of this journal on request.

## Ethical approval

Ethical approval is exempt in this case report publication. Informed written consent has been attained.

## Guarantor

Chrystal Calderon.

Robert Ramcharan.

## Funding

No funding/grants were received.

## Conflict of interest statement

None to declare.
